# Imaging of underground cavities with cosmic-ray muons from observations at Mt. Echia (Naples)

**DOI:** 10.1038/s41598-017-01277-3

**Published:** 2017-04-26

**Authors:** G. Saracino, L. Amato, F. Ambrosino, G. Antonucci, L. Bonechi, L. Cimmino, L. Consiglio, R. D.’ Alessandro, E. De Luzio, G. Minin, P. Noli, L. Scognamiglio, P. Strolin, A. Varriale

**Affiliations:** 1 Università degli studi di Napoli Federico II, Naples, Italy; 2 INFN sezione di Napoli, Naples, Italy; 3 TECNO-IN S.P.A., Naples, Italy; 4 INFN sezione di Firenze, Naples, Italy; 5 STRESS S.c.a r.l., Naples, Italy; 6 Università di Firenze, Naples, Italy; 7 Associazione Culturale Borbonica Sotterranea, Naples, Italy

## Abstract

Muography is an imaging technique based on the measurement of absorption profiles for muons as they pass through rocks and earth. Muons are produced in the interactions of high-energy cosmic rays in the Earth’s atmosphere. The technique is conceptually similar to usual X-ray radiography, but with extended capabilities of investigating over much larger thicknesses of matter thanks to the penetrating power of high-energy muons. Over the centuries a complex system of cavities has been excavated in the yellow tuff of Mt. Echia, the site of the earliest settlement of the city of Naples in the 8th century BC. A new generation muon detector designed by us, was installed under a total rock overburden of about 40 metres. A 26 days pilot run provided about 14 millions of muon events. A comparison of the measured and expected muon fluxes improved the knowledge of the average rock density. The observation of known cavities proved the validity of the muographic technique. Hints on the existence of a so far unknown cavity was obtained. The success of the investigation reported here demonstrates the substantial progress of muography in underground imaging and is likely to open new avenues for its widespread utilisation.

## Introduction

Muons are penetrating particles produced in Nature from the interactions of cosmic rays in the Earth’s atmosphere, with a spectrum extending to very high energies. These features allows us to extend the principle of standard X-ray radiography to the “muography” of very large thicknesses of matter (up to the order of 1 km of standard rock). Projective images of their density structure are provided through the determination of muon transmission, this in turn depends on the average densities and thicknesses of matter crossed by the muons.

To this purpose, the tracks of through-going muons are measured and the angular distribution of the reconstructed trajectories is compared to that expected in the absence of absorption, usually obtained from a dedicated data taking run with the detector pointing directly to the free sky. An expexted muon flux is then obtained from a map of the average density and traversed thicknesses as given by a Digital Terrain Model (DTM). A low density region, in particular a cavity, shows up in the data as an excess in the muon transmission.

The first steps in the field of muography occurred in 1955 with the determination of the rock overburden on a mountain tunnel from the measurement of the muon flux reduction^1^. In 1970 Louis Alvarez and collaborators performed a muography of the Chephren pyramid, searching for a hidden burial chamber^2^. The search was unsuccessful, but the feasibility of the technique was demonstrated. More recently the method has been applied in the study of the internal structure of volcanoes (see for example^3–9^ and references therein). Measurements or feasibility studies concerning muography applications in archaeology^10, 11^, mining^12^, tunnel searches^13, 14^, geological survey^15^ and nuclear waste detection^16^ have been reported in literature.

In the study reported here, the technique is used to investigate the underground cavities inside Mt. Echia in the city of Naples, with the additional aim of assessing its potentialities. The measurement was performed in the framework of the *METROPOLIS* project coordinated by *STRESS S*.*c*.*a r*.*l*. and was hosted by the cultural association *Borbonica Sotteranea*.

Mt. Echia (also called Pizzofalcone) hosted the ancient greek colony *Parthenope* founded in 8th century BC. That first settlement was subsequently called *Palepolis* (“old city”) when, in 5th century BC, its development led to the nearby foundation of *Neapolis* (“new city”) from which the denomination of Naples originates.

**Figure 1:**
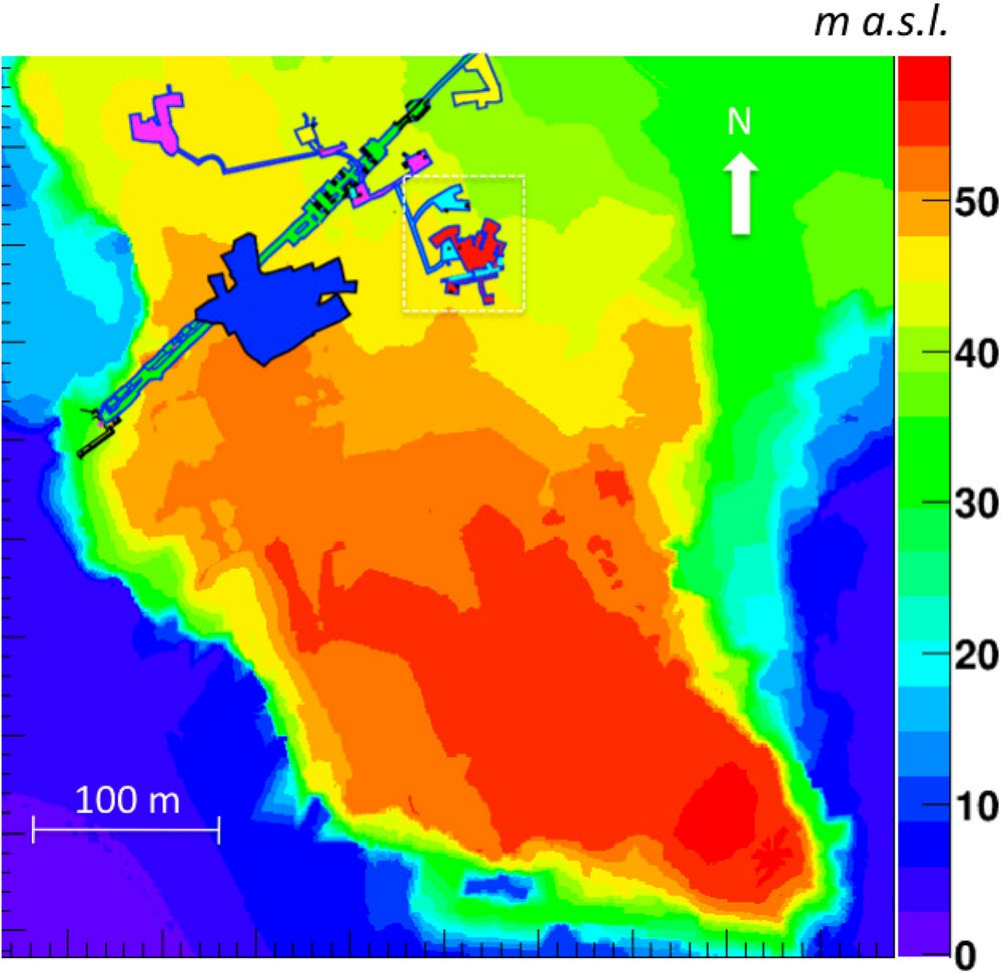
Digital Terrain Model (DTM) of Mt. Echia, with a resolution of 1 m in the horizontal x-y coordinates and 10 cm in the vertical z coordinate. The detector is located at 10 m a.s.l. within the dashed square, in the neighbourhood of the Bourbon Tunnel (straight line). The main underground structures located in the surrounding area are schematically shown. The map has been obtained by LIDAR observations elaborated by the following softwares: Terrasolid - Terrascan, LP360, RIEGL Riscan Pro, Golden Software - Surfer 12 and *root*.

**Figure 2:**
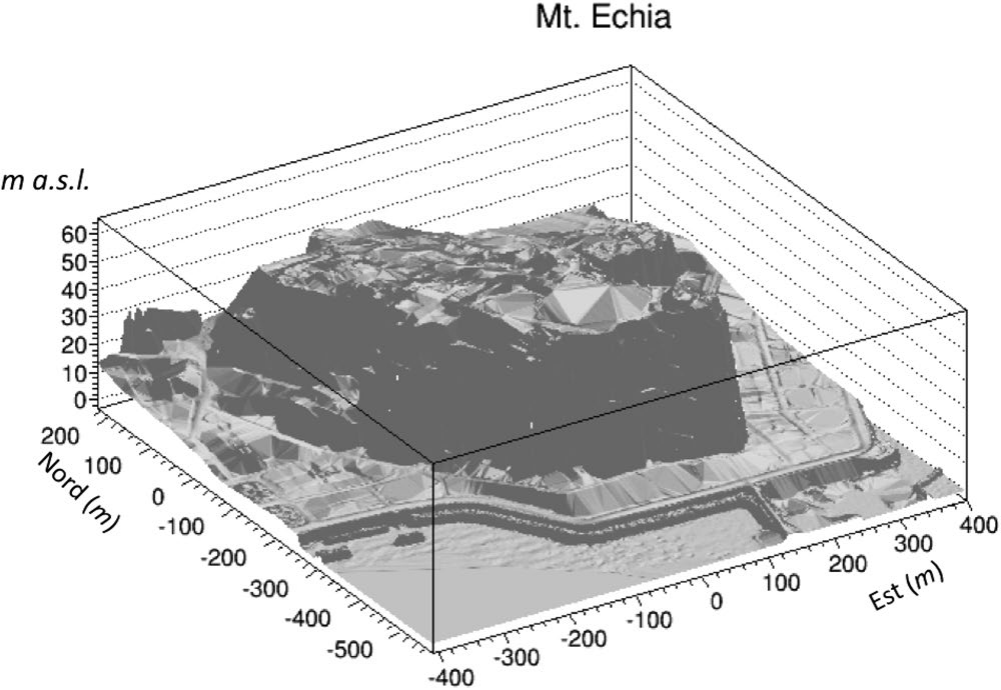
Three dimensional representation of the Digital Terrain Model (DTM) of Mt. Echia. The image was obtained using LIDAR observations elaborated by the following softwares: Terrasolid - Terrascan, LP360, RIEGL Riscan Pro, Golden Software - Surfer 12 and *root*.

The DTM of Mt. Echia is shown in Figs 1 and 2. Mt. Echia mainly consists of yellow tuff, a soft volcanic rock and its maximum altitude is about 60 m asl. In the course of history a very complex system of underground tunnels and cavities was excavated which only recently has become subject of systematic investigations.

The underground cavities served an astonishing variety of purposes, e.g. including that of a *mithraeum* (until the cult of Mithra was banned at the end of the 4th century AD). The tuff has been widely used as construction material: typically, tuff bricks were extracted from underground through a pit and the resulting cavities were then used for other purposes (e.g. water cisterns). In 1853 under the kingdom of Ferdinand II of the Bourbon House - *King of the Two Sicilies* - the so-called *Bourbon Tunnel* (see Fig. 1) was excavated through Mt. Echia for the purpose of connecting directly the Royal Palace and the military quarters, located on the opposite side. The *Bourbon Tunnel* has been restored in recent times and inserted in one of the archaeological underground itineraries, which include a large number of underground structures that have been rediscovered thanks to the ongoing underground exploration of Mt. Echia.

**Figure 3:**
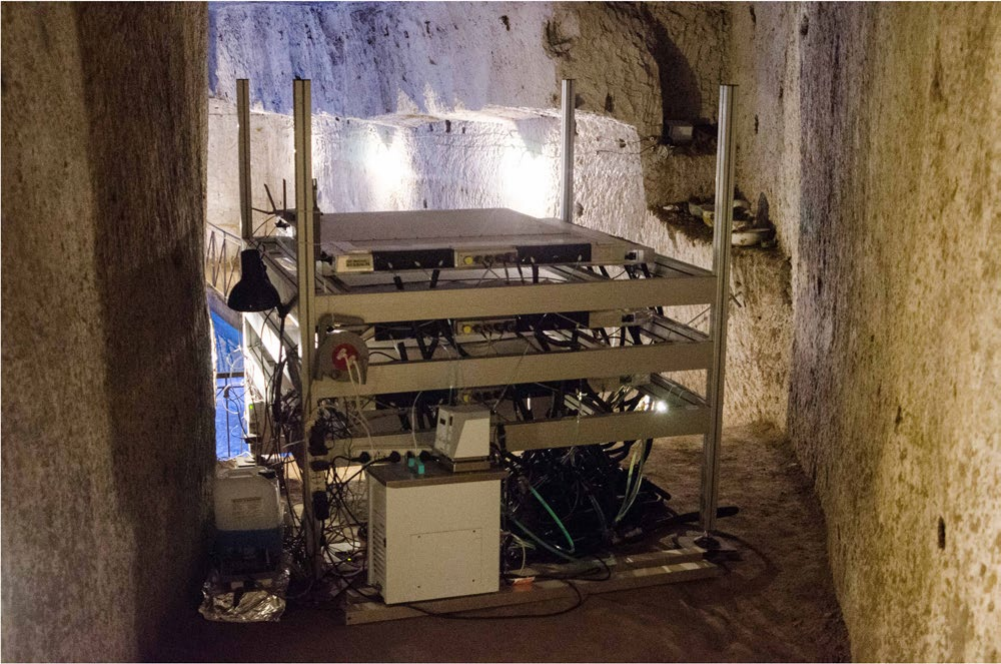
The detector in a recess of the cavity facing the water cistern.

**Figure 4:**
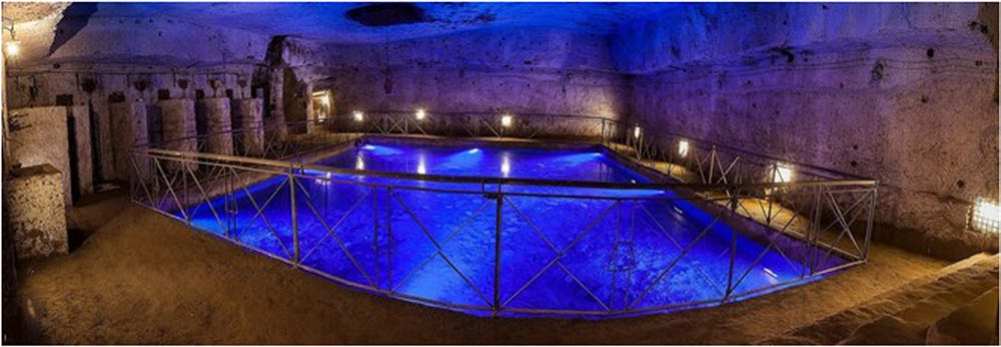
The water cistern, as seen from the detector location.

The muon detector is shown in Fig. 3 and is described in the next section. It was accommodated in a small recess of a cavity containing an underground water cistern (Fig. 4) and connected to the *Bourbon Tunnel* (see Fig. 1), so that it was rather easily accessible. Above the detector there are known cavities, suitable for test the technique as a first step for possible further investigations.

## The muon detector

**Figure 5:**
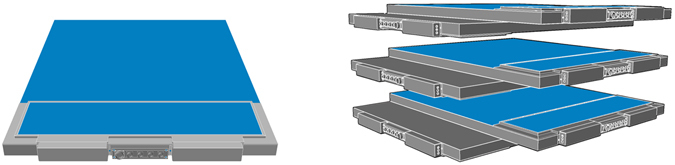
Left: Sketch of the aluminium shell of a detection plane, consisting in 64 scintillator bars and the Front-End Electronics. Right: Sketch of the muon telescope, consisting of a vertical sequence of three modules, mounted horizontally on a support (not shown) and evenly spaced in the vertical direction (z-axis).

The muon detector is an upgraded version of the MU-RAY detector, developed for volcano muography^17–19^. The main upgrades concern mechanics and the power supply system. Two MU-RAY half planes are assembled in a single aluminium shell together with the Front-End Electronics, forming a detection plane of about 1 m^2^ with handles installed that can be easily transported (see Fig. 5). The detection planes are mounted on the measurement site and the cabling is performed using few round-shape connectors. Also the electric power supply has been simplified and all the needed voltages, including the Silicon photomultipliers bias voltage, are produced by the front-end boards starting from a single 6 V supply.

The MU-RAY basic sensitive element to crossing muons are plastic scintillator bars of triangular cross-section.

**Figure 6:**
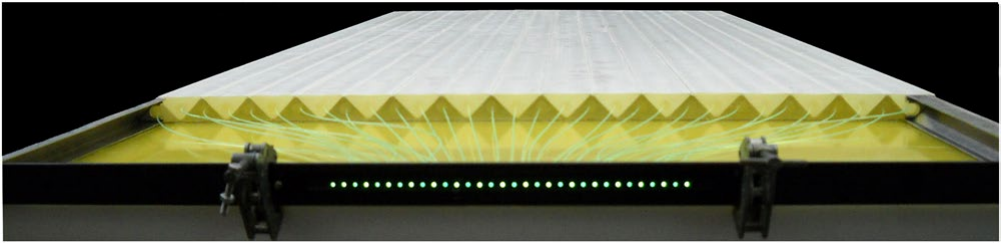
A half plane of 32 scintillator bars and 32 wavelength shifting fibres placed in a connector. The connector is optically coupled to 32 Silicon photomultipliers, mounted on a printed circuit board, not shown in the picture. Two half planes mounted side by side form a detection plane.

Each bar has a length of 107 cm, a 3.3 cm base and a height of 1.7 cm. They are extruded with a central 2 mm hole through which a 1 mm diameter wavelength shifter fibres (*BICRON BCF92*) is threaded. The bars have a *TiO*_2_ coating (0.25 mm thick) that increases the internal reflectivity and screens them from outside light sources^20^. The light signals produced by muons in the plastic scintillator bars are conveyed to photosensors by the fibres. The photosensors are based on the solid-state Silicon Photo-Multiplier (SiPM) technology, that allows for photo-electron counting. The photosensors are produced by Hamamatsu Photonics (MPPC S12825-050P). A detection plane consists of two half planes (each with 32 adjacent bars alternatively turned upside down) covering an area of about 1 m^2^. Each 32 fibre bundle is optically coupled to a printed circuit board hosting 32 Silicon photomultipliers (see Fig. 6) thanks to a custom made connector.

A full sized module consists of a pair of superimposed planes (see Fig. 5) orthogonally orientated, that provide the x and y coordinates of the muon impact point with a spatial resolution of about 2 mm. Each plane is equipped with two Front-End Electronics (FEE) boards based on the ASIC chip EASIROC^21^. The passage of a penetrating charged particle through the detector provides a fast digital signal each time a muon crosses a detection plane. A time coincidence of the signals from the six planes triggers the data acquisition. The analogue information related to the amount of energy released by muons in each bar is digitised by Analog to Digital Converters (ADC), acquired by Data Acquisition (DAQ) boards and stored for offline reconstruction of the muon tracks. The maximum possible acquisition rate of the detector was about 270 Hz.

The setup installed at Mt. Echia consisted of a vertical sequence of three modules, mounted horizontally on a support and evenly spaced in the vertical direction (z-axis) as shown in Fig. 5. In the setup used for the measurement reported here, the distance between the bottom and the top module was about 50 cm. In this configuration the muon track angular resolution was 4 mrad in each projection with a maximum observable field of view from the Zenith of about 63°. During the whole data taking, a control system maintained the SiPM temperature at about 18 °C with a stability of about 0.01 °C, using an hydraulic circuit connected to a chiller.

## Data taking

Approximatively 14 × 10^6^ events were acquired during two different periods of 26 days in total under Mt. Echia. Data taking was performed in runs of about 1 hour, storing all the ADC outputs whenever a trigger was generated. Pedestal events were also acquired to calculate offsets and noise values.

**Figure 7:**
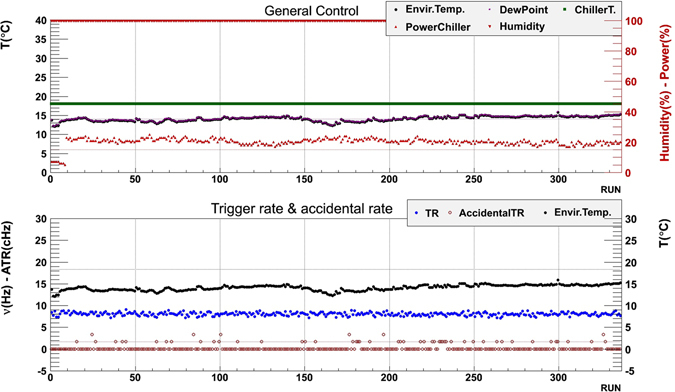
Stability of the main parameters that monitor the status of the detector during the Mt. Echia data tacking. Top: Environmental temperature, dew point temperature, chiller temperature and power, relative humidity. Bottom: Trigger rate, accidental rates, environmental temperature. The drawing was obtained using the software *root*.

**Figure 8:**
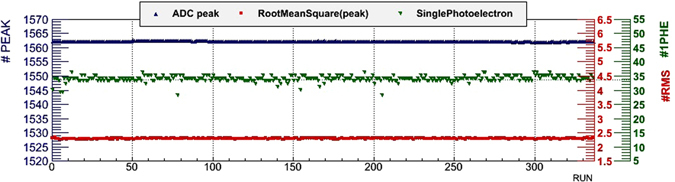
Stability of the main parameters acquired during pedestal runs, for one random channel, during the Mt. Echia data tacking: mean and RMS value of the ADC peak (blue triangles and red squares), the ADC mean value corresponding the single photoelectron response (green triangles). The drawing was obtained using the software *root*.

For each run the trigger rate, the accidental trigger rate, the dark rates of the SiPMs and the offsets (*pedestals*) of the ADC outputs were monitored, as well as environment and instrument parameters such as environmental temperature, relative humidity and dew point temperature, chiller temperature, electrical power consumption, currents and voltages supplied to the SiPMs. All these parameters were found to be stable, as shown in Figs 7 and 8.

Thanks to the excellent intrinsic resolution of the SiPMs, after pedestal subtraction from the ADC spectra the signals corresponding to a single photoelectron (SPE responses) were determined. The SPE responses allow to quantify the average number of photoelectrons produced by a muon in each scintillator bar and to monitor in time the gain stability of the system. The SPE responses were found to be stable during all the data taking periods. The trigger rate was constantly at around 8.1 Hz.

A free sky calibration sample of about 12 × 10^6^ events was acquired in a surface laboratory. In this run, a 25 mm thick iron layer and a 10 mm thick iron lead layer were inserted between the second and the third detector modules from the top. These layers introduced a cut of about 100 MeV on the energy of muons crossing the detector. The trigger rate during the calibration run was 77 Hz.

## Muon track reconstruction

Muon tracks are reconstructed event by event independently in the x-z and y-z projections, where x and y represent the coordinates in the horizontal plane and z is the vertical coordinate.

The normalised energy deposited in a scintillator bar is defined as the pedestal subtracted ADC value divided by the SPE. Clusters are formed by considering one or more neighbouring bars with a significant deposit. The normalized energy of a cluster is the sum of the energies of the bars forming the cluster while the x and y coordinates of the cluster are the energy weighted averages of the bars coordinates.

In each projection a successful fit of clusters belonging to the three planes defines a candidate track. The track candidate with the highest value of the minimum cluster energy is defined as the best candidate track. The three-dimensional best candidate track associates the best candidate tracks in the two projections.

Quality cuts were further introduced to improve the purity of the sample. The *χ* ^2^/ndf of the fit was required to be less than 5 and the minimum energy of the clusters associated to the track was required to be greater than 4. In addition, we selected events containing only single muon tracks by requiring the absence of other clusters with a normalised energy greater than 4, and we restricted to 2 the maximum number of bars in a given cluster. This sample of *golden tracks* has a standard deviation of ∼2 mm in the track fit residual distribution.

**Figure 9:**
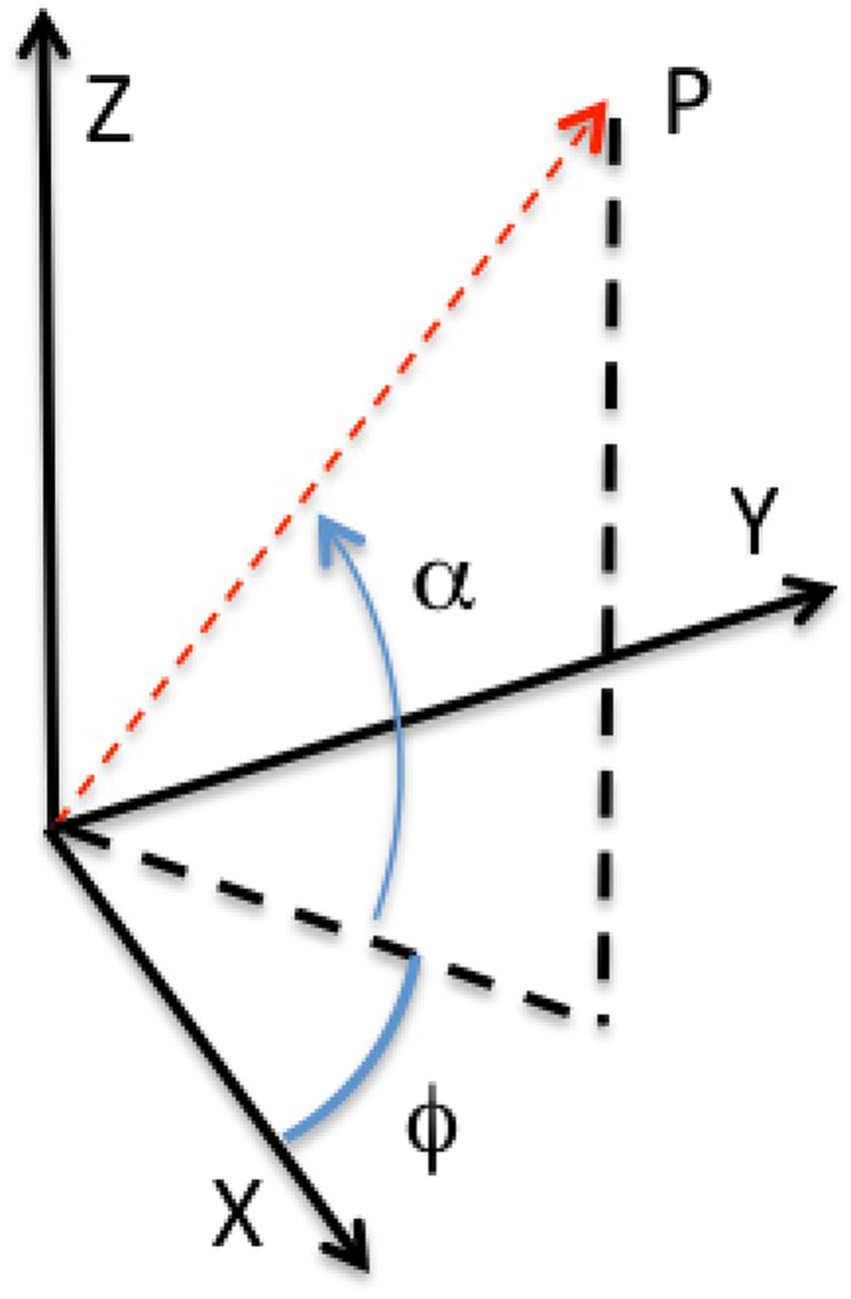
Definition of the elevation angle *α* and the horizontal angle *ϕ*.

Events with a golden tracks in both views constitute a 3D muon track, with elevation angle *α* and horizontal angle *ϕ* defined as in Fig. 9. Their values are plotted event-by-event in a bi-dimensional histogram, typically with a 3° × 3° binning.

## Muon transmission

Muon transmission is defined as the ratio between the muon flux reaching the detector divided by the flux impinging on the surface before crossing the thickness of rock under investigation. The presence of a cavity is thus identified by an excess in the measured transmission relative to the expected transmission in its absence.

**Figure 10:**
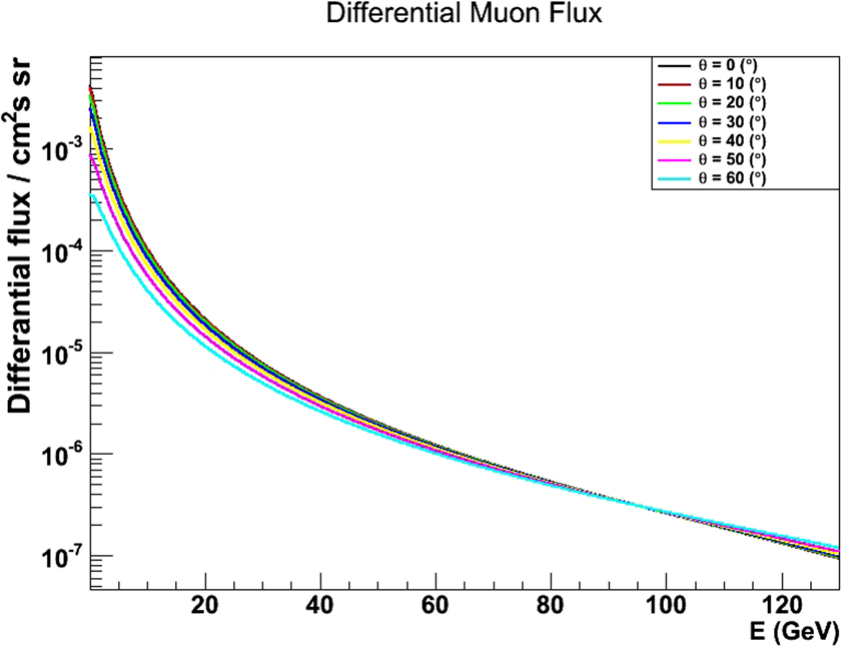
Differential muon flux distribution as a function of the muon energy, for different zenith angles, as given in ref. 22. The drawing was obtained using the software *root*.

The number of muons ${N}^{u}(\rho ,\alpha ,\varphi )$ which in the absence of internal structure (such as cavities) are expected to be recorded underground in any given angular bin around $(\alpha ,\varphi )$ in a data taking time ${\rm{\Delta }}{T}^{u}$ can be written as

1 $${N}^{u}(\rho ,\alpha ,\varphi )={\rm{\Delta }}{T}^{u}\cdot {S}_{eff}(\alpha ,\varphi ){\int }_{{E}_{min}}^{\infty }{\rm{\Phi }}(\alpha ,\varphi ,E)\,{\rm{d}}E$$

where ${S}_{eff}(\alpha ,\varphi )$ is the so-called effective area of the detector and ${\rm{\Phi }}(\alpha ,\varphi ,E)$ is the differential muon flux as a function of the muon energy *E*. In particular, the differential muon flux was measured by the *ADAMO* experiment^22^ in the zenith angle range 0°–80° and energies in the range 100 MeV–130 GeV. Some of the differential flux curves are shown in Fig. 10.

The lower limit ${E}_{min}(\rho ,\alpha ,\varphi )$ of the integral in eq. 1 is the minimum energy that muons must have in order to cross the rock and reach the detector. It depends of average rock density *ρ* and of the rock thickness, the latter being fixed by the DTM for any given (*α*, *ϕ*)^23^. *E*_*min*_ is evaluated from models that parametrise the muon energy loss in matter^24^. The detector was placed inside a chamber of approximatively cubic shape with sides approximately 3 m long. In order to avoid a systematic overestimation of the rock thickness this empty volume has been taken in account.

**Figure 11:**
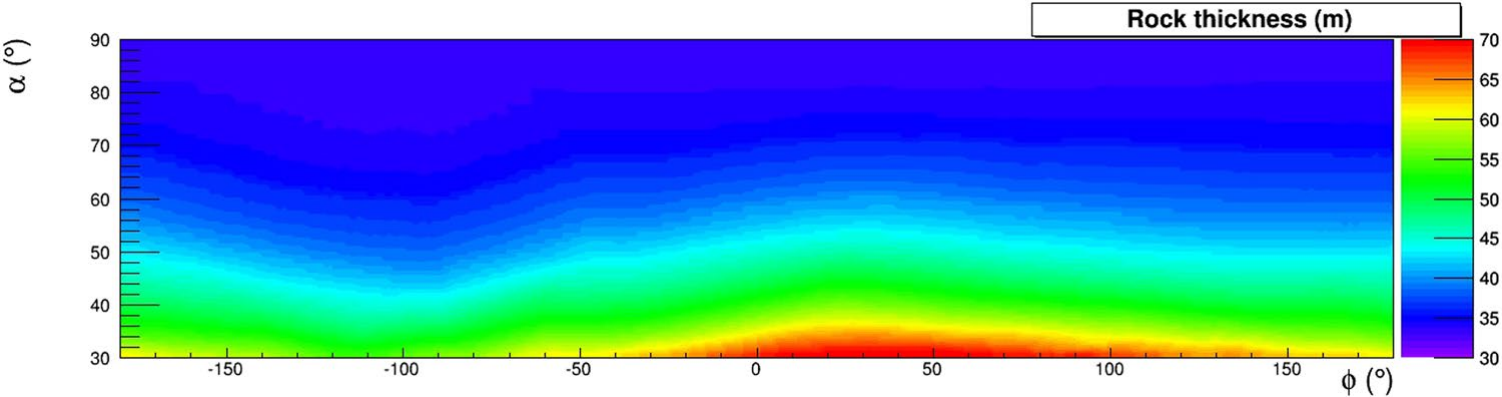
Rock thickness *d*(*α*, *ϕ*) crossed by muons reaching the detector under the hypothesis that no cavities are present, evaluated using the Digital Terrain Model. The drawing was obtained using the software *root*.

**Figure 12:**
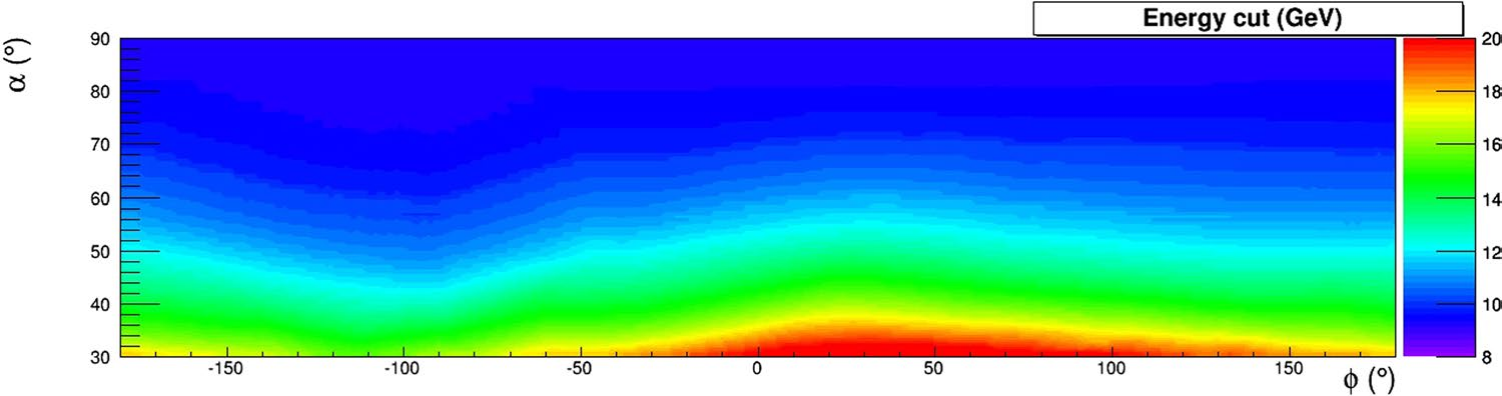
Minimum energy ${E}_{min}(\rho ,\alpha ,\varphi )$ required for a muon to cross the rock overburden, whose thickness is shown in Fig. 11, evaluated for an uniform average rock density *ρ* = 1.4 g/cm^3^. The drawing was obtained using the software *root*.

The colour scale in the two-dimensional (*α*, *ϕ*) histogram shown in Fig. 11 shows the rock thickness surrounding the detector. The minimum energy ${E}_{min}(\rho ,\alpha ,\varphi )$ in the absence of internal structure that appears in eq. 1 is shown using an appropriate colour scale in the two-dimensional (*α*, *ϕ*) histogram shown in Fig. 12, for an uniform average rock density *ρ* = 1.4 g/cm^3^.

The effective detector area does not depend significantly on the muon energy and can be written as follows:

2 $${S}_{eff}(\alpha ,\varphi )=S\cdot A(\alpha ,\varphi )\cdot {\varepsilon }_{trig}(\alpha ,\varphi )\cdot {\varepsilon }_{DAQ}({\nu }_{trig})\cdot {\varepsilon }_{an}(\alpha ,\varphi )$$

where *S* is the sensitive area of the detector, $A(\alpha ,\varphi )$ is its geometrical acceptance, ${\varepsilon }_{trig}(\alpha ,\varphi )$ is the trigger efficiency, ${\varepsilon }_{DAQ}({\nu }_{trig})$ is the data acquisition efficiency (that depends of the trigger rate ${\nu }_{trig}$) and ${\varepsilon }_{an}(\alpha ,\varphi )$ is the analysis efficiency.

Similarly, the number of free-sky muons expected in a time interval ${\rm{\Delta }}{T}^{fs}$ can be written as:

3 $${N}^{fs}(\alpha ,\varphi )={\rm{\Delta }}{T}^{fs}\cdot {S}_{eff}^{fs}(\alpha ,\varphi ){\int }_{{E}_{0}}^{\infty }{\rm{\Phi }}(\alpha ,\varphi ,E)\,{\rm{d}}E$$

where *E*_0_ is the minimum energy required to detect muons in the laboratory, estimated at about 100 MeV and supposedly independent of (*α*, *ϕ*) in the angular range under study.

The expected ratio between the underground and the free sky samples can be written as

4 $$\frac{{N}^{u}(\rho ,\alpha ,\varphi )}{{N}^{fs}(\alpha ,\varphi )}=\frac{{\rm{\Delta }}{T}^{u}\cdot {S}_{eff}^{u}(\alpha ,\varphi )}{{\rm{\Delta }}{T}^{fs}\cdot {S}_{eff}^{fs}(\alpha ,\varphi )}\frac{{\int }_{{E}_{min}}^{\infty }{\rm{\Phi }}(\alpha ,\varphi ,E){\rm{d}}E}{{\int }_{{E}_{0}}^{\infty }{\rm{\Phi }}(\alpha ,\varphi ,E){\rm{d}}E}$$

With good approximation it can be assumed that the geometrical acceptance, the trigger efficiency, and the analysis efficiency are the same in the two data samples and cancel out in the ratio obtaining:

5 $$\frac{{N}^{u}(\rho ,\alpha ,\varphi )}{{N}^{fs}(\alpha ,\varphi )}=C\cdot \frac{{\int }_{{E}_{min}}^{\infty }{\rm{\Phi }}(\alpha ,\varphi ,E){\rm{d}}E}{{\int }_{{E}_{0}}^{\infty }{\rm{\Phi }}(\alpha ,\varphi ,E){\rm{d}}E}$$

where C is the calculable constant

6 $$C=\frac{{\rm{\Delta }}{T}^{u}}{{\rm{\Delta }}{T}^{fs}}\cdot \frac{{\varepsilon }_{DAQ}({\nu }_{fs})}{{\varepsilon }_{DAQ}({\nu }_{u})}$$

**Figure 13:**
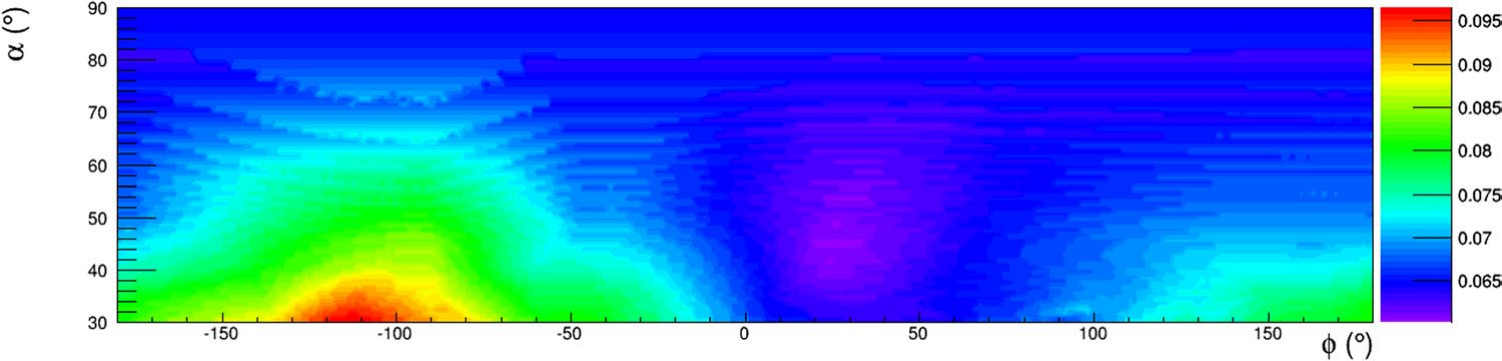
The expected transmission. The drawing was obtained using the software *root*.

The quantity

7 $$T(\rho ,\alpha ,\varphi )=\frac{{\int }_{{E}_{min}}^{\infty }{\rm{\Phi }}(\alpha ,\varphi ,E){\rm{d}}E}{{\int }_{{E}_{0}}^{\infty }{\rm{\Phi }}(\alpha ,\varphi ,E){\rm{d}}E}$$

represents the expected transmission (see Fig. 13).

Its value is obtained using an integration limit ${E}_{min}(\rho ,\alpha ,\varphi )$ evaluated as specified above, under the hypothesis that no cavities are present.

From eq. 5, the measured transmission is calculated as

8 $${T}^{m}(\alpha ,\varphi )=\frac{1}{C}\frac{{N}^{u}(\alpha ,\varphi )}{{N}^{fs}(\alpha ,\varphi )}$$

**Figure 14:**
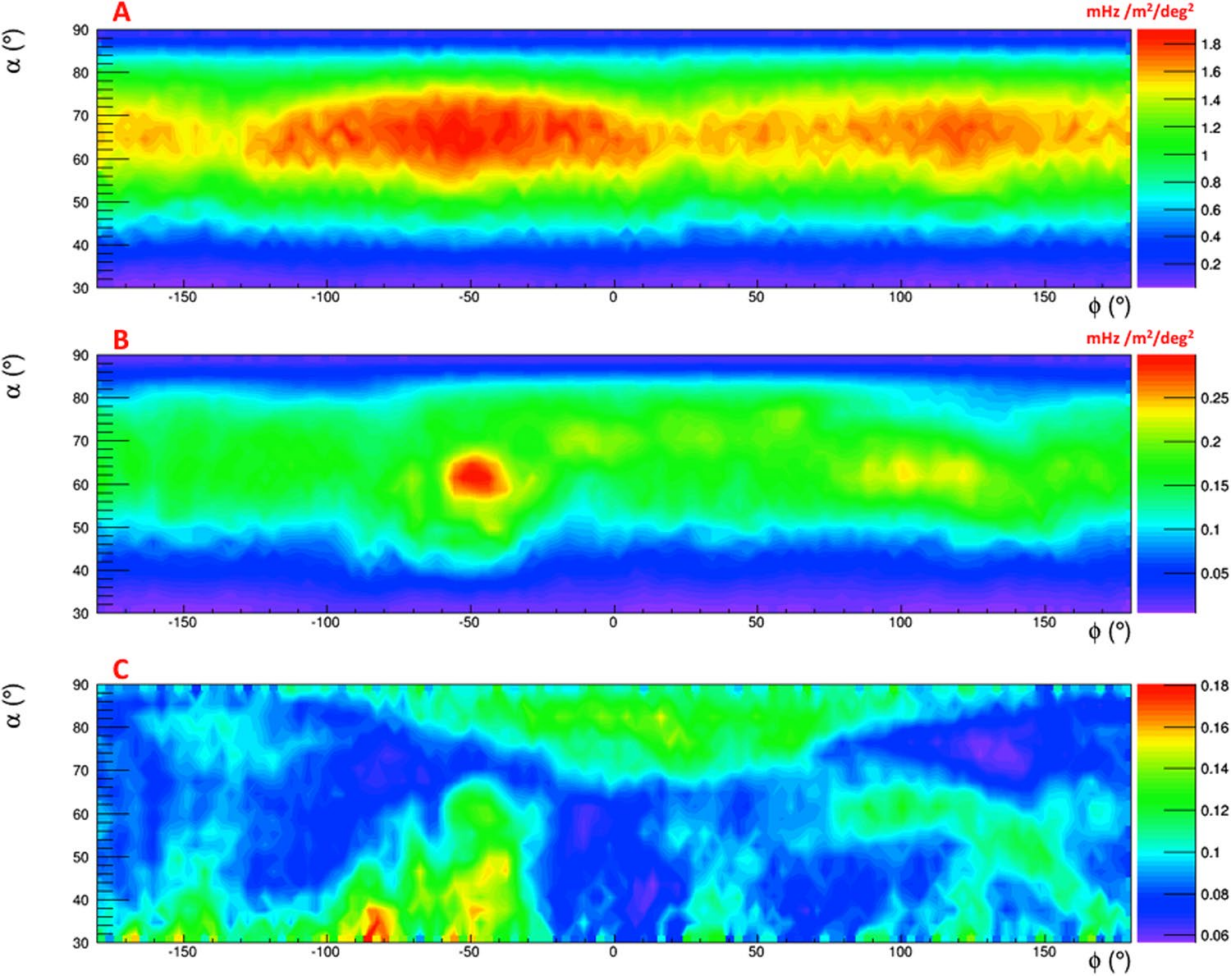
(**A**) Rate of free-sky muons measured in laboratory. (**B**) Rate of underground muons measured under Mt Echia. (**C**) Measured transmission. The drawing was obtained using the software *root*.

In Fig. 14 the rate of free-sky muons measured in laboratory, the rate of underground muons measured under Mt. Echia and the measured transmission are shown as function of the elevation and horizontal angles.

The relative transmission *R*(*ρ*, α, *ϕ*) is defined as the ratio between the measured and the expected transmission:

9 $$R(\rho ,\alpha ,\varphi )=\frac{{T}^{m}(\alpha ,\varphi )}{T(\rho ,\alpha ,\varphi )}$$

The relative transmission equals unity in case no internal structure is present and the correct rock density *ρ* is inserted.

**Figure 15:**
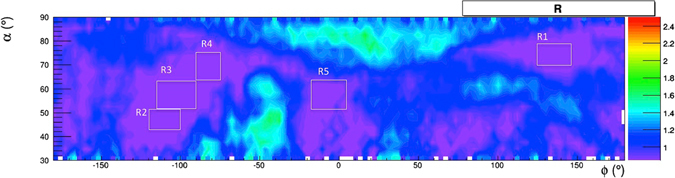
The relative transmission R($\rho ,\alpha ,\varphi$) evaluated with *ρ* = 1.4 g/cm^3^. Regions with values higher than 2.5 have been saturated at this value. Also shown are five regions, where minimal relative transmission values have been selected to evaluate the best estimation of the density. The drawing was obtained using the software *root*.

The density of a grain of yellow tuff is in the range of 2.4 ÷ 2.5 g/cm^3^ and the porosity is in the range of 50% ÷ 60%. Typical values of the density of dry yellow tuff in the Naples area are in the range 1.0 ÷ 1.2 g/cm^3^, but the actual density depends of the water contents^25, 26^. The path length of muons in the rock is evaluated from the DTM, so that the presence of buildings is accounted for by an “effective density” higher the real rock density. As a first step in the evaluation of the expected transmission, hence of the relative transmission, we take an effective density *ρ* = 1.4 g/cm^3^. Figure 15 gives the map of the relative transmission R(*ρ*, *α*, *ϕ*), The smoothing algorithm *contour*4 of the *root* analysis software tool is applied^27^.

## Measurement of the rock density

A measurement of the effective density can be performed using angular regions where minimal relative transmission values are present. One may assume that in these regions no cavities have been encountered by muons and the value *R* = 1 is expected.

**Figure 16:**
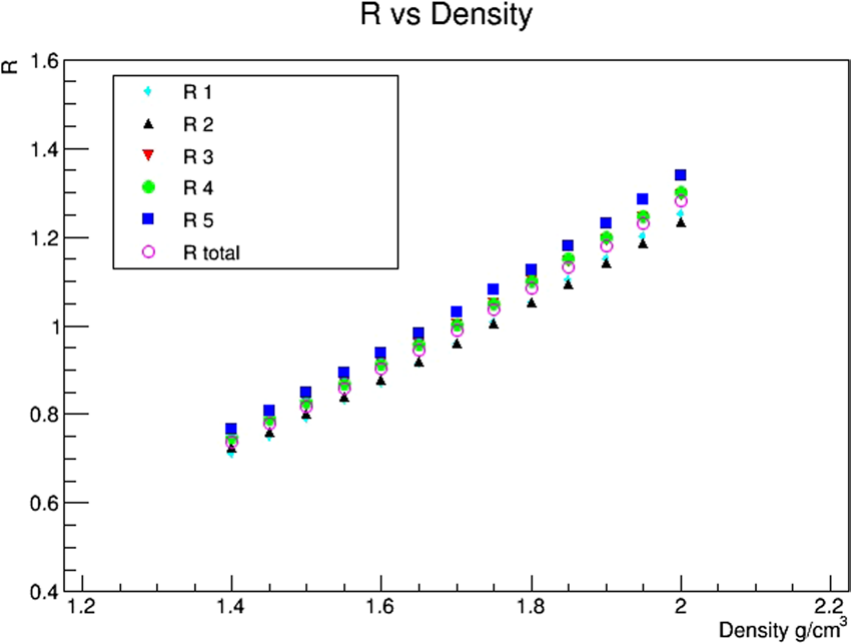
Relative transmissions in the five regions *R*_1_–*R*_5_ (separately and in total) as functions of *ρ*. The drawing was obtained using the software *root*.

Five regions (*R*_1_–*R*_5_) were defined (see Fig. 15) and the relative transmission value for different density values was evaluated. Figure 16 gives the relative transmissions in the five regions and in their total, as functions of *ρ*, using values R < 0.85.

The density was evaluated by interpolating the line in R = 1 giving a density *ρ* ^*m*^ = 1.71 g/cm^3^.

Repeating the procedure using value of R < 0.75 gives a difference less than 3%.

The standard deviation of the average value obtained from separate fits to the five regions is *σ*_*ρ*_ = 0.013 g/cm^3^. Another contribution to the error in the density measurement comes from the uncertainties in the rock thickness from the DTM (1 m in *x* and *y*, 10 cm in *z*) and from the error in the detector position. The entire density measurement procedure was thus repeated changing rock thickness up to 5 m, in steps of 1 m. The trend is approximatively linear and the error is 0.04 g/cm^3^ for 1 meter uncertainty. Adding in quadrature the two contributions, one obtains an error of 0.042 g/cm^3^ (2.5%) in the density measurement. The statistical contribution was evaluated and is negligible.

In principle, through a nonlinear, effect a deviation of *ρ* from reality may fake a non uniformity that could erroneously be attributed to a fake internal structure. Actually, with a good approximation, ${E}_{min}(\rho ,\alpha ,\varphi )$ depends linearly of the rock density. However, the nonlinearity of muon spectrum (Fig. 10) reflects in a nonlinear dependence of the expected transmission of ${E}_{min}(\rho ,\alpha ,\varphi )$, hence also of *ρ*, resulting in an apparent not uniformity in the transmission. Quantitatively, a deviation within the above mentioned (*a priori*) density range of yellow tuff in the Naples area (±20%) was found to induce a fake disuniformity smaller than ∼4%. Such a fake disuniformity would limit the sensitivity to the detection of cavities e.g. to a 1.5 m size over 50 m rock thickness. This sensitivity limit is further reduced when considering the above uncertainty on the measured density.

## Observed structures

**Figure 17:**
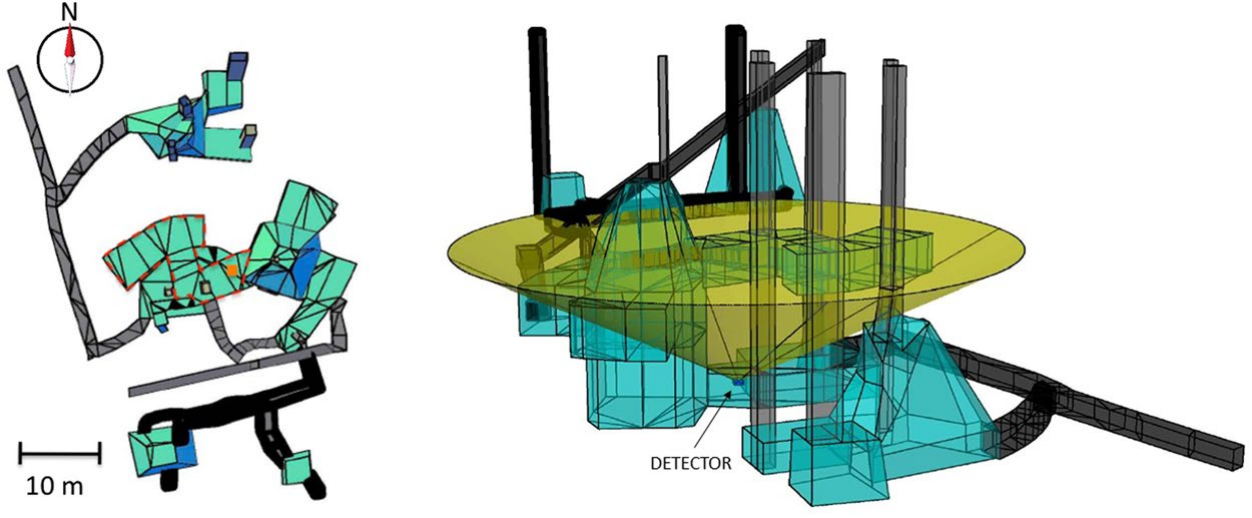
Left: top view of the known structures in the neighbourhood of the detector. The orange box shows the detector position, below the room indicated by the dashed line. Right: 3D view. The cone, with ∼120° of aperture, represents the angular acceptance of the detector. The drawings were obtained using the software Autodesk AutoCAD 2015.

Figure 17 shows the known cavities in the neighbourhood of the detector. The total rock thickness in the vertical direction above the detector amounts to about 40 m. Figure 17 shows the structures within the geometrical acceptance of the detector, a cone of ∼60° semi-aperture from Zenith.

**Figure 18:**
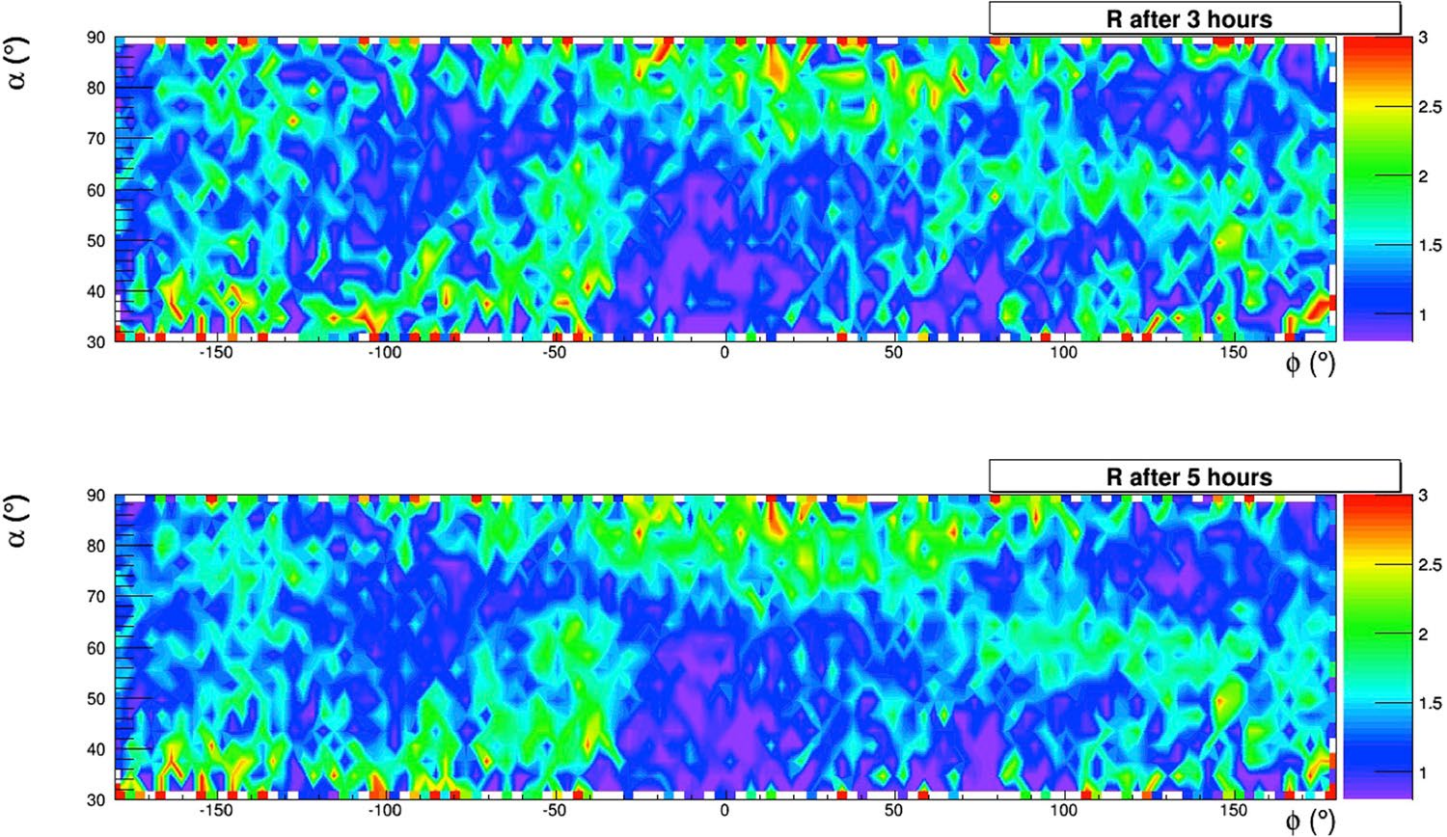
Time sequence of muographies obtained in 5 hours of data taking. The drawing was obtained using the software *root*.

**Figure 19:**
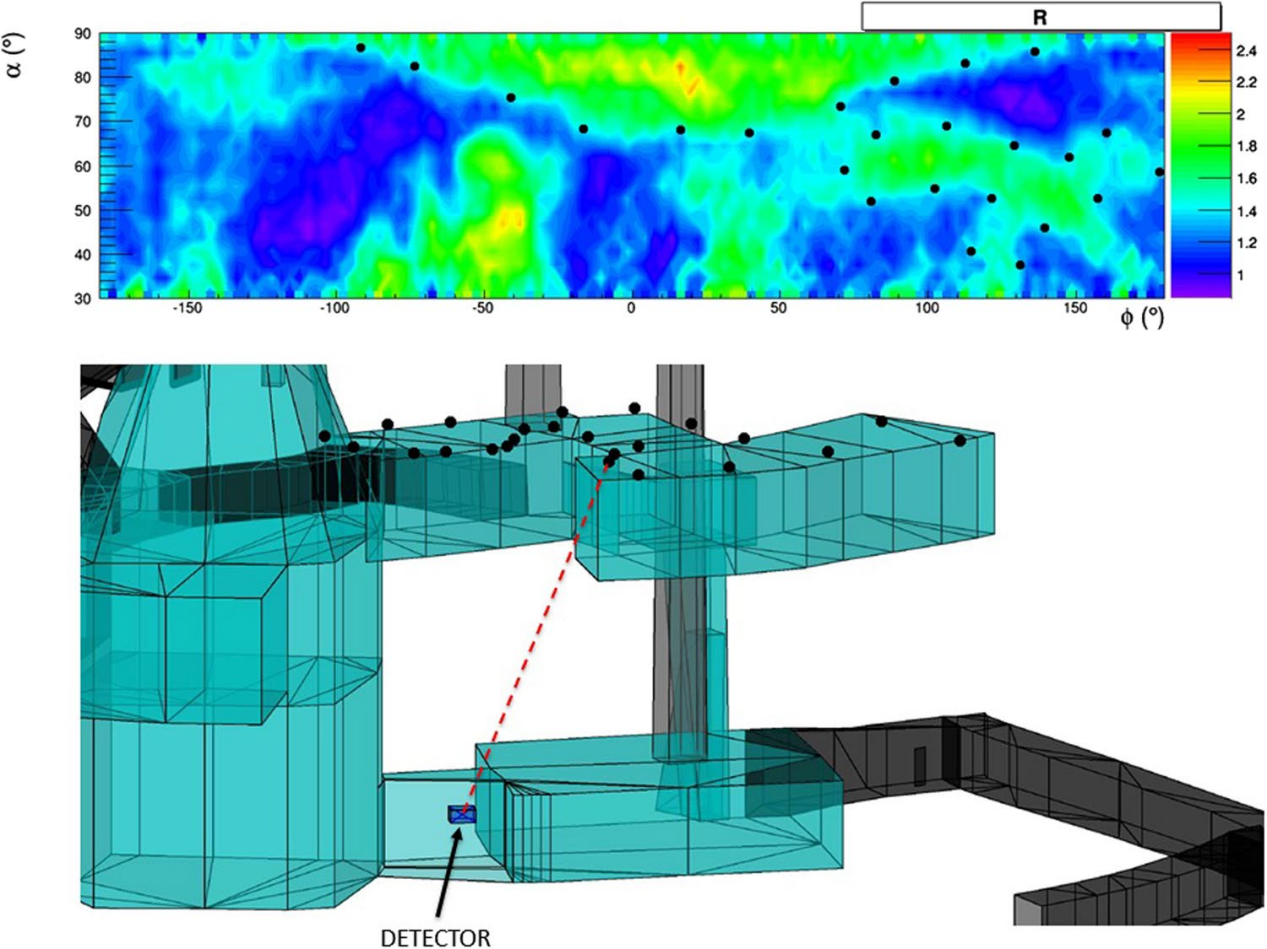
Top: Relative transmission R($\rho ,\theta ,\varphi )$ evaluated with the best estimate of the tuff density *ρ* = 1.71 g/cm^3^; the black dots lie on the contour of one of the structures which are observed. Bottom: Projection of the black dots in the top figure at z = 25 m, corresponding to the ceiling of a known structure. The drawings were obtained using the software *root* and Autodesk AutoCAD 2015.

Muographic evidence of such structures emerges already after some hours of data taking: Fig. 18 shows how the angular map of the relative transmission takes shape during the first 5 hours. The top part of Fig. 19 shows the map (using angular coordinates) that was obtained in 26 days of data taking. Both maps are evaluated with the measured density *ρ* ^*m*^ = 1.71 g/cm^3^.

We focus on one of the structures which are observed in the top part of Fig. 19. This structure is identified by the black dots lying on its contour, as defined by the level R = 1.71 of the relative transmission. We seek a correspondence with a sequence of known cavities located above the detector, all of them having a height of about 4.5 m and with the ceiling at 25 m altitude a.s.l. The 3D image given in the bottom part of Fig. 19 thus includes (red dots) the projections at z = 25 m of the black dots lying on the contour of the above structure in the muography. The correspondence of the muographic image to a real structure is demonstrated once the slanted view of the cavities from the detector location is taken into account.

**Figure 20:**
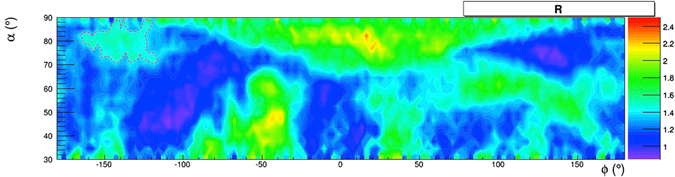
A structure evidenced by the muographic image and with contour shown by a dashed line, without any correspondence among the known structures. The drawing was obtained using the software *root*.

Most of the other structures that are *muographically* visible point to known structures. A hint of the presence of a hidden cavity is given by the structure indicated by a dashed line in Fig. 20. This structure could be a partially filled cavity which geologists, currently investigating the system of cavities in the neighbourhood of the Galleria Borbonica, suspect could be present in that direction.

## Conclusions

A new generation muon detector exploiting current state of the art technologies was installed in a cavity in Mt. Echia in Naples under about 40 m of yellow tuff overburden, and took data in a 26 days pilot run. The average rock density was measured, improving the imaging sensitivity. The muography technique was validated by the identification of known internal structures, already evident after a few hours of data taking. Hints of the existence of so far unknown cavities have been obtained. Further measurements will be performed from different detector locations, in order to stereoscopically reconstruct in space the observed cavities and possibly confirm the hints for structures which have not yet been discovered by other conventional means.

The amount of matter overburden at Mt. Echia (about 40 m) is of general interest for a wide range of applications including archaeology, geological surveys and civil engineering. By exploiting present electronic technologies, compact muon detectors can be built that are capable of providing useful data in a matter of weeks and to analyse them quasi online by standard means from remote locations. The successful observations reported here may thus have a broader impact on the long awaited establishment of muography as a standard technique.
